# Effects of virtual reality on chest tube removal pain management in patients undergoing coronary artery bypass grafting: a randomized clinical trial

**DOI:** 10.1038/s41598-024-53544-9

**Published:** 2024-02-05

**Authors:** Zahra Dalir, Fatemeh Seddighi, Habibollah Esmaily, Mohammad Abbasi Tashnizi, Elahe Ramezanzade Tabriz

**Affiliations:** 1 Department of Medical Surgical Nursing, School of Nursing and Midwifery, Mashhad University of Medical Sciences, Azadi Square, Shahid Dr. Kharazmi Educational Complex, PO Box 9177949025, Mashhad, Iran; 2 Department of Biostatistics, School of Health, Mashhad University of Medical Sciences, Mashhad, Iran; 3 Department of Cardiac Surgery, Imam Reza Hospital, Mashhad University of Medical Sciences, Mashhad, Iran

**Keywords:** Cardiac device therapy, Pain, Cardiology, Diseases

## Abstract

The pain associated with chest tube removal (CTR) is one of the significant complications of cardiac surgery. The management of this pain is recognized as a vital component of nursing care. The application of distraction techniques using virtual reality (VR) is an effective and straightforward non-pharmacological approach to alleviate pain. This study aimed to determine the impact of VR technology on the management of pain caused by CTR following coronary artery bypass grafting (CABG). This randomized clinical trial was conducted on 70 patients undergoing CABG at Imam Reza and Qaem hospitals in Mashhad, Iran, in 2020. The patients were randomly divided into two groups of 35. For the intervention group, a 360-degree video was played using VR glasses 5 min before the CTR procedure. The pain intensity was measured before, immediately after, and 15 min after CTR, using the Visual Analogue Scale. Also, the Depression Anxiety and Stress Scale-21 (DASS-21), and the Rhoten Fatigue Scale (RFS) were used to evaluate intervention and control groups before the CTR procedure. The collected data was analyzed using statistical tests, such as Chi-square, independent t-test, and Mann–Whitney test. The patients were homogeneous in terms of stress, anxiety, and fatigue levels before CTR, and they did not show any significant differences (*P* > 0.05). The average pain intensity score of patients in the intervention group significantly decreased immediately and 15 min after CTR, compared to the control group (*P* < 0.001). Given the positive impact of VR distraction on the severity of pain associated with CTR in patients undergoing CABG, this technique can serve as an effective, accessible, and cost-efficient non-pharmacological approach for managing pain in these patients.

**Trial registration:** This study was registered in the Iranian Registry of Clinical Trials (code: IRCT20190708044147N1; approval date, 08/26/2019).

## Introduction

Cardiovascular disease (CVD) is the most common chronic disease worldwide. Among various types of CVDs, coronary artery disease has the highest incidence rate and is the leading cause of mortality associated with CVD^1^. The global burden of disease (GBD) in 2019 reports that CVD is responsible for 18.5 million deaths worldwide, accounting for approximately 31% of total deaths^2^. In Iran, the prevalence of this disease shows a rising trend, and it is estimated to be the cause of nearly 50% of all deaths annually^3^. Coronary artery bypass grafting (CABG) is an important surgical procedure, typically performed for patients with severe coronary artery stenosis, who are unresponsive to other forms of treatment. Every year, hundreds of millions of people undergo CABG globally. In Iran alone, 25,000 open-heart surgeries are performed annually, with 50–60% of these procedures being CABG-related^4^.

Patients undergoing CABG often experience significant postoperative pain and chest tube removal (CTR) is the most painful procedure performed for patients following CABG^5^. This procedure often leads to anxiety and discomfort, tension and worry, as well as physical changes, such as an increase in heart rate, blood pressure, breathing rate, and muscle tone^6,7^. Since the use of a chest tube, followed by its subsequent removal (CTR) is a crucial medical procedure after CABG, management of the resulting pain, especially during removal, is of utmost importance to minimize complications^8^. While there is substantial evidence supporting various methods for postoperative pain management, ensuring adequate pain relief remains a significant challenge in the field of nursing^9,10^. Patients are often dissatisfied with repeated injections and insufficient doses of ineffective analgesics during the first three days following surgery^11,12^.

In the past few decades, pharmacological methods have been more commonly used to control the pain of CTR^13–16^, but recently, guidelines have deemed pharmacological methods alone as insufficient and have recommended the incorporation of non-pharmacological methods^17^. Non-pharmacological methods are often easily accepted and well-received by patients. Additionally, they do not cause any undesirable complications or side effects that are typically associated with pharmaceutical interventions^18^. Therefore, numerous efforts have been undertaken to employ non-pharmacological techniques, such as thermotherapy (heat and cold therapy), relaxation strategies, breathing exercises, music therapy, and aromatherapy to alleviate the pain of CTR^13,18–20^.

Distraction techniques serve as highly effective non-pharmacological methods in diminishing the perception of pain. When painkillers prove to be insufficiently effective and are accompanied by side effects, virtual reality (VR) distraction emerges as a viable alternative for pain management. This method is not only effective, easy to implement, and cost-efficient, but is also associated with minimal side effects^21^. With its distinctive capabilities in gaming, film, and simulation domains, VR effectively diverts one’s attention^22^. Through the use of VR glasses and smartphones, this technology allows users to select and interact with 360-degree videos or images of real environments that have been previously captured. This immersive experience offers users the advantage of altering their surroundings in a virtual setting^23^.

So far, VR has found a multitude of applications within the healthcare sector, including the treatment of phobias and anxiety disorders, cognitive and physical rehabilitation, management of both acute and chronic pain, and procedures, such as blood sampling, intravenous insertions, vaccinations, and burn treatments in both children and adults. Additionally, VR provides support to cancer patients during painful procedures and chemotherapy sessions and is utilized in the treatment of eating disorders and obesity, as well as in surgical training^24–28^. The results of the studies have been different based on the population and the clinical intervention of the study. However, the quality of the evidence is limited and statistically heterogeneous. Therefore, before the development of virtual reality in the clinic, there is a need for more and higher quality studies. Since no study was found on the intensity of pain caused by CTR in patients undergoing CABG using this technology, and considering the advances in VR and non-pharmacological pain relief methods, including VR distraction, in this study, we aimed to determine the impact of VR technology on pain management during CTR following CABG.

## Methods

### Participants

This randomized clinical trial, involving two groups, was carried out at Imam Reza and Qaem hospitals in Mashhad, Iran, during 2020. The study population comprised all patients who were referred for CABG and met the inclusion criteria for participation in this study. The patients (n = 70) were selected among CABG patients admitted to the intensive care unit (ICU) and post-ICU departments of cardiac surgery at Imam Reza and Qaem hospitals.

The criteria for inclusion in the study were as follows: (1) being in the age range of 18–70 years; (2) undergoing CABG surgery and having a pericardial or mediastinal tube; (3) possessing sufficient vision and hearing capabilities (ability to see images with or without glasses and hear sounds from a distance of at least half a meter); (4) having sufficient alertness according to the Richmond Agitation-Sedation Scale (RASS) (score 0, indicating that the patient is alert and calm) to determine the intensity of pain^29^; (5) having minimum reading and writing literacy; (6) being an Iranian; (6) not having conditions, such as delirium, dementia, or severe depression; (7) not having received injectable pain medications within four hours prior to the CABG surgery; and (8) not being connected to a ventilator. On the other hand, the criteria for exclusion from the study were as follows: (1) changes in the patient’s hemodynamic status; (2) requiring medical or surgical interventions; and (3) the need to use painkillers and sedatives while the procedure is being performed.

### Sample size

Since we found no studies similar to our research examining the impact of VR application on CTR pain management after CABG surgery, the sample size was determined based on the results of a pilot study. This pilot study involved 10 participants in each group and utilized the mean comparison formula. The maximum value derived from these computations was subsequently adopted as the sample size for this study. The sample size formula is as follows:

$$n = \frac{{\left( {Z_{{1 - \frac{\alpha }{2}}} + Z_{1 - \beta } } \right)^{2} \left( {\delta_{1}^{2} + \delta_{2}^{2} } \right)}}{{\left( {\mu_{1} - \mu_{2} } \right)^{2} }}$$

A sample size of 30 individuals per group was calculated at a confidence level of 99% and a test power of 90%. To increase the level of certainty, an additional 15% was added to this estimate.

### Randomization

In this study, patients were recruited using a non-probability convenience sampling method. The patients were randomly allocated to the groups to mitigate the potential for information dissemination among the participants. They were divided into two groups via block randomization with a block size of two. The patients were randomly assigned to the intervention group (n = 35) and the control group (n = 35). The intervention group was exposed to VR distraction using a VR headset, while the control group received routine care. In the block randomization method, initially, 35 random numbers were selected between 0 and 9 from a table of random numbers. If the selected number was even, “AB” was written in the list, and if it was odd, “BA” was written. Next, based on the order of patients’ arrival, they were assigned to either group A (intervention) or group B (control)^30^. To prevent the spread of information in the ICU and post-ICU departments of cardiac surgery, measures were taken to ensure that the environment of the participants was separate from other patients during the intervention.

### Scales of measurement and variables

In this study, the data collection tools included a demographic information questionnaire, the Visual Analogue Scale (VAS), the Depression Anxiety and Stress Scale-21 (DASS-21), and the Rhoten Fatigue Scale (RFS).

#### Demographic information questionnaire

This questionnaire contained 16 questions related to the individual characteristics of the patients, including age, sex, marital status, education level, height, weight, drug and tobacco use, sleep duration in the last 24 h, location, size, and length of the chest tube, history of cardiac surgery, and history of chest tube placement.

#### Visual analogue scale (VAS)

This scale, as one of the most reliable and widely used pain measurement tools, was designed to measure pain intensity in patients. It is a 10-cm scale, with 0 on the left indicating an absence of pain, and 10 on the right denoting the highest level of pain one can conceive. A score of 1–3 indicates mild pain, 4–7 indicates moderate pain, and 8–10 indicates severe pain. The validity and reliability of this scale have been confirmed in several studies, including studies by Gift, Shaban et al., and Hassanzadeh et al.^18,31,32^.

#### Depression, anxiety and stress scale-21 (DASS-21)

In 1995, Lovibond and Lovibond developed a self-report questionnaire, known as DASS. The short version of this scale consists of 21 items, with each item (score range, 0–21) representing a psychological construct. On this scale, depression, anxiety, and stress are each assessed by a set of seven questions, and the questions are rated on a four-point Likert scale (0: ‘Did not apply to me at all’, 1: ‘Applied to me to some degree’, 2: ‘Applied to me to a considerable degree’, and 3: ‘Applied to me very much’). Generally, DASS-21 is a short version of the primary scale, with scores for stress and anxiety ranging from 0 to 21 in each category. The final score for each subscale is doubled to ascertain the severity of the symptoms^33,34^. The reliability and validity of this questionnaire have been confirmed by Asghari et al., who reported a reliability coefficient of 0.94^35^. In our study, the reliability of this scale was also confirmed using the Cronbach’s alpha method (α = 0.81).

#### Rhoten fatigue scale (RFS)

This scale, which was designed to measure the level of fatigue in patients, is easily understood by patients. It is a 10-cm scale, where 0 on the left side signifies no fatigue, and 10 on the right side represents the maximum level of fatigue. A score of 1–3 on the scale signifies mild fatigue, a score of 4–7 represents moderate fatigue, and a score of 8–10 indicates severe fatigue^36,37^.

### Interventions

After selecting the patients, the researcher explained the objectives of the research to the patients, and they were asked to complete the demographic information questionnaire, as well as informed consent forms. Additionally, depression, anxiety, and stress were assessed with the DASS-21 scale, and fatigue was evaluated with the RFS for intervention and control groups before the CTR procedure. Pain management depends on the psychological factors of patients such as anxiety, depression, and fatigue^38^, and according to the necessity of homogeneity of groups and determining the level of these factors, these variables were investigated in the control and intervention groups. It is worth mentioning that the chest tube used for the intervention was the first tube that could be removed according to the physician’s orders.

In the intervention group, the VR distraction technique was implemented using the VR Shinecon G04BS Virtual Reality Glasses manufactured by China. Initially, the patient was provided with a detailed explanation about the method of intervention. Subsequently, a selection of five 360-degree videos, each featuring natural landscapes with a minimum duration of 5 min, was presented to the patients to choose from. Once the patients chose their preferred video, a smartphone was placed into the headset. The selected video was then prepared for viewing through the VR application. Before wearing the headset, the pain intensity was measured and recorded with the VAS.

**Figure 1:**
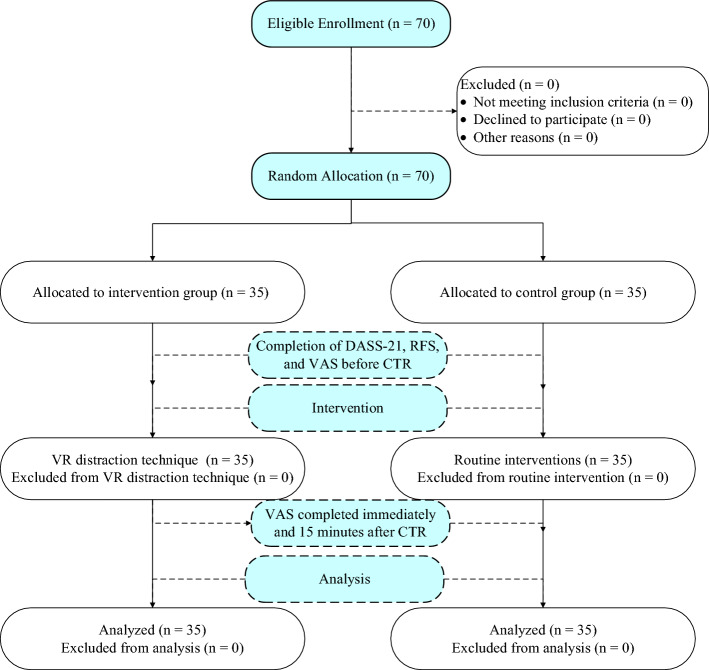
The CONSORT flow diagram of the study.

Five minutes before the CTR procedure, the headset was placed on the patient’s head, and the video was played. After the fifth minute, a nurse trained in an intensive care unit carried out CTR by the standard procedure with the supervision of a CABG surgeon. Subsequently, the video playback was halted, and the VR glasses were taken off from the patient’s eyes. The pain intensity was measured and recorded immediately and 15 min after CTR. In the control group, routine care for CTR was performed. In routine care, the patient was encouraged to perform the Valsalva maneuver (deep breathing and holding it until the CTR) to distract the patient and reduce lung collapse. The intensity of pain was assessed in the control group during the same intervals as the intervention group, using the aforementioned scales (Fig. 1).

### Statistical analysis

Once the data was collected and the accuracy of data entry was confirmed, statistical analysis was conducted using both descriptive and inferential methods in IBM SPSS Version 25. To characterize the participants in the study, descriptive statistics were measured, including absolute and relative frequency distributions for qualitative variables and mean and standard deviation (SD) calculations for quantitative variables. Subsequently, the normality of the distribution of quantitative variables was determined by Shapiro–Wilk test.

The homogeneity of the two groups in terms of normally distributed quantitative variables was assessed using the independent t-test. For non-normally distributed quantitative variables and ranked variables, the Mann–Whitney test was employed. Moreover, to compare the three stages of analysis (before, immediately after, and 15 min after the intervention) within each group, repeated measures ANOVA was used for normally distributed variables, while Friedman’s test was employed for variables that were not normally distributed. For within-group comparisons between the pre- and post-intervention stages, the paired t-test was performed for normally distributed variables, while the Wilcoxon test was employed for variables that were not normally distributed. In all the tests, the confidence interval was set at 95%, and a P-value of less than 0.05 was considered statistically significant.

### Ethics approval and consent to participate

The study was approved by the Ethics Committee of Mashhad University of Medical Sciences, Mashhad, Iran (Code: IR.MUMS.NURSE.REC.1398.027) and registered in the Iranian Registry of Clinical Trials (IRCT20190708044147N1, 08/26/2019). The study protocol followed the Ethical Guidelines of the Declaration of Helsinki. Prior to initiating the study, the objectives and methodology of the research were clearly explained to both the officials and the research participants. Informed consent was obtained from the participants before the study after assuring them about the safety of the interventions, the confidentiality of their information, and their right to withdraw from the study at any time. The research units were monitored in all stages of the study, and their well-being was ensured until the end of the study.

## Results

In the present study, a total of 70 patients undergoing CABG, who met the inclusion criteria, were allocated to the control (n = 35) and intervention (n = 35) groups. The demographic and clinical information of the patients is presented in Table 1. The results of data analysis showed no significant difference between the two groups before the intervention, and the groups were homogeneous (*P* > 0.05) (Table 1).

**Table 1:** Sociodemographic and clinical characteristics of participants.

Characteristics	Control group (n = 35)	Experimental group (n = 35)	Statistical tests		
			*F*/*χ*^2^, Z, t	df	*p*
Age (years), mean (SD)	52.3 ± 11.5	54.5 ± 8.3	0.9*	68*	0.367*
Gender n (%)					
Male	24 (68.6)	24 (68.6)	0.0**	1**	0.001**
Female	11(31.4)	11(31.4)			
Body mass index, mean (SD)	26.3 ± 3.6	26.9 ± 3.7	0.6*	68*	0.541*
Drug addiction n (%)					
Yes	8 (22.9)	5 (14.3)	0.9**	1**	0.356**
No	27 (77.1)	30 (85.7)			
Sleep duration before CRT (hour), mean (SD)	5.2 ± 1.6	5.4 ± 1.2	− 1.0***	–	0.310***
Chest tube insertion site					
Mediastinal	8 (22.9)	9 (25.7)	0.1**	1**	0.780**
Pericardium	27 (77.1)	26 (74.3)			
Days of chest tube inserted (hour), n (%)					
24	9 (25.7)	7 (20.0)	− 0.8***	–	0.435***
48	22 (62.9)	22 (62.9)			
72	4 (11.4)	6 (17.1)			
Chest tube size (Fr), n (%)					
24	5 (14.3)	3 (8.6)	− 1.3***	–	0.179***
26	10 (28.6)	7 (20.0)			
28	15 (42.9)	17 (48.6)			
29	5 (14.3)	8 (22.9)			
Chest tube length (cm), n (%)					
14	9 (25.7)	3 (8.6)	− 1.3***	–	0.196***
16	12 (34.3)	14 (40.0)			
18	9 (25.7)	13 (37.1)			
20	5 (14.3)	5 (14.3)			

*Independent t-test.

**Chi-square test.

***Mann–Whitney.

Before the intervention, the participants reported moderate stress, severe anxiety, and moderate fatigue. However, they were homogeneous in terms of stress, anxiety, and fatigue and did not show any significant differences (*P* > 0.05) (Table 2).

**Table 2:** Comparison of stress, anxiety and fatigue intensity scores among groups before CTR.

Variable mean (SD)	Control group (n = 35)	Experimental group (n = 35)	Statistical tests		
			Z, t	df	*p*
Stress	13.3 (5.1)	14.2 (6.4)	− 0.3*	–	0.740*
Anxiety	19 (4.7)	19.6 (5.8)	0.5**	68**	0.619**
Fatigue	4 (1.8)	3.4 (1.6)	− 1.4**	68**	0.161**

*Mann–Whitney.

**Independent t-test.

Before CTR, the results of independent t-test did not show any significant difference between the pain intensity scores of the patients in the two groups (*P* = 0.156). However, the results of the Mann–Whitney test showed that the difference in the pain intensity scores of the patients decreased by 1.5 ± 1.3 in the intervention group and increased by 0.3 ± 1.3 in the control group immediately after CTR compared to the pre-intervention stage. The test results showed this difference to be significant (*P* < 0.001), and the effect size was found to be very large (d = 1.389). Also, 15 min after CTR, the difference in the pain intensity score reduced by 3.7 ± 1.3 in the intervention group and by 2.1 ± 1.7 in the control group compared to the pre-intervention stage. The Mann–Whitney test showed this difference to be significant (*P* < 0.001), and the effect size was very large (d = 1.089) (Table 3).

**Table 3:** Comparison of pain intensity scores among groups before and after CTR.

Pain intensity scores mean (SD)		Control group (n = 35)	Experimental group (n = 35)	Statistical between groups tests		
				Z, t, χ2	df	*p*
Before CTR		5.7 (1.5)	6.2 (1.5)	1.4*	68*	0.156*
Immediately before CTR		5.9 (1.6)	4.6 (1.4)	− 3.4**	–	0.001**
15 min after CTR		3.5 (1.6)	2.5 (1.4)	− 3.0**	–	0.003**
The difference before with immediately after CTR		0.3 (1.3)	− 1.5 (1.3)	− 4.9**	–	< 0.001**
The difference between before and 15 min after CTR		− 2.1 (1.7)	− 3.7 (1.3)	− 3.9**	–	< 0.001**
The difference between immediately after and 15 min after CTR		− 2.4 (1.1)	− 2.2 (1/0)	− 0.8**	–	0.414**
Statistical within group test	*F*/*χ*^2^	62.3***	49.2***			
	df	2***	2***			
	*p*	< 0.001***	< 0.001***			

*Independent t-test.

**Mann–Whitney.

***Friedman test.

## Discussion

This study aimed to determine the impact of VR technology on pain management during CTR following CABG. The comparison of intervention and control groups revealed that the pain intensity scores significantly decreased immediately and 15 min after CTR in the intervention group, compared to the control group. This result indicates that VR is effective in alleviating pain immediately after CTR. However, it should be noted that 15 min post-CTR, a significant reduction in pain was observed in both groups; this suggests that the intensity of pain experienced by patients can gradually diminish over time following CTR. In a study by Özkan et al., it was found that the use of distraction through VR glasses had a significant effect on pain management during CTR in patients undergoing thoracostomy with the diagnosis of pneumothorax without surgery. In this study, a notable decrease in pain was observed following CTR in the intervention group^39^. Other studies, such as those conducted by Orhan et al.^39^, Menekli et al.^41^ and Karaveli Çakır et al.^42^, have also found that the use of VR is effective in managing patients’ pain; these findings align with the results of the current study^40–42^. Conversely, in a study by Laghlam et al.^5^, while the use of VR was well-tolerated by patients following cardiac surgery, it did not yield positive results in managing the pain and anxiety associated with CTR when compared to an equimolar mixture of oxygen and nitrous oxide (Kalinox^®^) used on the second day after cardiac surgery. Laghlam et al. proposed that this difference could be due to the sedative or amnestic effects of Kalinox^®^; they also reported the rapid onset of action of this combination, which was easy to use^5^.

In the present study, it was found that patients experience moderate to severe pain during CTR, suggesting an increase compared to previous levels. Also, the pain intensity score decreased by 90% in the intervention group immediately after CTR compared to the pre-intervention stage. In a study by Ford et al., it was reported that the intensity of pain experienced by the burn dressing change group decreased by 60% when they watched a selected VR movie^23^; this finding aligns with the results of the current study. Moreover, a systematic review on the role of VR in pain management in inpatient settings showed that 67% of studies reported a significant reduction in pain^43^. In another study by Kodvavi et al., assessing the impact of VR on patient pain and anxiety during the preoperative period, VR significantly diminished pain both during and after the procedure^44^.

In other studies that investigated the effectiveness of VR on pain relief, it was found that VR was significantly effective in managing pain during burn physiotherapy and needle-related procedures. However, it did not yield significant results for minor surgical procedures and burn wound treatments^45^. Also, using VR glasses for 10 min during the episiotomy procedure reduced pain and increased the satisfaction of pregnant mothers^40^. The effectiveness of distraction through VR as an accessible, cost-effective, and non-invasive technique for pain and anxiety during fine needle aspiration (FNA), colonoscopy, and endoscopic surgery was also reported^41,46,47^. Additionally, the use of VR with D3 films has been found to significantly reduce the pain associated with dressing changes in the VR group, compared to the control group^48^. Meanwhile, a study by Glennon et al.^49^, comparing the effects of distraction with VR and watching TV on the level of pain and anxiety in patients undergoing bone marrow biopsy and aspiration, found no reduction in pain or anxiety in the VR group, whereas, in the control group watching TV, there was a reduction in pain and anxiety. The visual and audio content was the same in both groups, and the only difference between the groups was the use of headsets in the VR group. Glennon's study results contradict our findings; a potential reason for this discrepancy could be the type of procedure performed on the patients. It appears that the VR distraction technique does not alleviate pain associated with needle-related procedures, such as lumbar puncture^49,50^.

Despite the clear differences in study type, population, implementation methods, hardware and software equipment, and clinical scenarios across various studies, a significant decrease was observed in the patient’s pain intensity scores in both the current study and other studies. Despite the mentioned differences in methods in studies, the consistency in the results could be attributed to the mechanism of VR effectiveness. This technology creates a form of distraction, shifting the patient’s focus away from the painful stimulus. As a result, it significantly reduces the patient’s perception of pain^51^. Generally, the findings of this study indicate that the use of VR, as a novel distraction technique, can effectively reduce the patient’s perception of pain during the CTR procedure. In terms of pain intensity, given that the control and intervention groups were homogeneous in all background and confounding variables in this study, it can be inferred that the findings of this study are likely attributable to the effects of the intervention. The effectiveness of VR can be elucidated through the lens of the cognitive theory of emotion. This model posits that our perception of pain is structured psychologically and elicits an emotional response. The degree of attention we pay to a harmful or painful stimulus serves as a gauge for our perception of pain. According to this model, the attention capacity of the brain is limited, and the brain must focus on the painful stimulus to perceive pain. Therefore, the perception of pain is limited when people's attention is diverted from the stimulus. This mechanism can result in a decrease in attention to pain by competing with the sensory stimuli of pain, thereby leading to reduced sensitivity to pain. In the realm of VR technology, the patient’s attention is captivated by a simulated computer world. Given the brain’s limitations in processing information, this technique triggers a flood of multi-sensory information to be processed simultaneously; this in turn diminishes the patient’s focus on painful signals, thereby alleviating the sensation of pain^52–54^.

### Limitations

First, the variations in pain perception and tolerance thresholds among individuals, as well as the unique psychological differences of the research participants, may have influenced their responses. Second, the potential for bias exists in this study due to the absence of blinding in the intervention. Finally, with the rapid advancement of technology and the evolution of VR equipment, using more up-to-date and higher quality equipment could potentially lead to a more effective reduction in pain levels. However, in this study, cost-effectiveness was prioritized, leading to the use of readily available and less expensive equipment.

### Implications for nursing practice

In accordance with the nursing standards, prioritizing patient comfort and pain relief is essential. Nurses, equipped with an understanding of the pathophysiology of pain and its treatment methods, should strive to manage the patients’ pain in line with the nursing process. The simultaneous use of simple, effective, and low-cost non-pharmacological methods, such as VR, for pain management can reduce the occurrence of unwanted physiological consequences of severe pain.

## Conclusion

The application of VR distraction techniques can be effective in reducing the intensity of pain caused by CTR in patients undergoing CABG. The findings of this study can improve the understanding of treatment and healthcare teams regarding the application of VR techniques. As a non-pharmacological and beneficial technique, VR can be effectively used in managing pain associated with procedures, such as CTR, in patients following CABG.

## Data Availability

The datasets used and/or analyzed during the current study are available from the corresponding author upon reasonable request.

## Abbreviations

CTR: Chest tube removal
VR: Virtual reality
CABG: Coronary artery bypass grafting
CVD: Cardiovascular disease
ICU: Intensive care unit
RASS: Richmond agitation-sedation scale
VAS: Visual analogue scale
DASS-21: Depression anxiety and stress scale-21
RFS: Rhoten fatigue scale
SD: Standard deviation
FNA: Fine needle aspiration

## References

[1] Virani SS, Alonso A, Aparicio HJ, Benjamin EJ, Bittencourt MS, Callaway CW (2021). Heart disease and stroke statistics-2021 update: A report from the American Heart Association. Circulation..

[2] Zhang Y, Lin C, Liu M, Zhang W, Xun X, Wu J, Li X, Luo Z (2023). Burden and trend of cardiovascular diseases among people under 20 years in China, Western Pacific region, and the world: An analysis of the global burden of disease study in 2019. Front. Cardiovasc. Med..

[3] Farhangi MA, Najafi M (2018). Dietary inflammatory index: A potent association with cardiovascular risk factors among patients candidate for coronary artery bypass grafting (CABG) surgery. Nutr. J..

[4] Babajani S, Babatabar Darzi H, Ebadi A, Mahmoudi H, Nasiri A (2014). The effect of foot reflexology massage on the level of removal after open heart surgery. Iran. J. Crit. Care Nurs..

[5] Laghlam D, Naudin C, Coroyer L, Aidan V, Malvy J, Rahoual G, Estagnasié P, Squara P (2021). Virtual reality vs. Kalinox® for management of pain in intensive care unit after cardiac surgery: A randomized study. Ann. Intensive Care..

[6] Mokadem NME, Shimaa E, Ibraheem S (2017). Cold application and breathing exercises to reduce pain and anxiety during chest tube removal. Am. J. Nurs. Sci..

[7] Mohammadi N, Pooria A, Yarahmadi S, Tarrahi MJ, Najafizadeh H, Abbasi P, Moradi B (2018). Effects of cold application on chest tube removal pain in heart surgery patients. Tanaffos..

[8] Puntillo KA, Max A, Timsit J-F, Vignoud L, Chanques G, Robleda G (2014). Determinants of procedural pain intensity in the intensive care unit. The Europain® study. Am. J. Respir. Crit. Care Med..

[9] Dehghani Z, Keikhaei A, Yaghoubinia F, Keykha A, Khoshfetrat M (2018). Impact of pain management algorithm on pain intensity of patients with loss of consciousness hospitalized in intensive care unit: A clinical trial. Med. Surg. Nurs. J..

[10] Ouellette C, Henry S, Turner A, Clyne W, Furze G, Bird M, Sanchez K, Watt-Watson J, Carroll S, Devereaux PJ, McGillion M (2019). The need for novel strategies to address postoperative pain associated with cardiac surgery: A commentary and introduction to “SMArTVIEW”. Can. J. Pain..

[11] de Andrade ÉV, Haas VJ, de Faria MF, Dos Santos Felix MM, Ferreira MBG, Barichello E, da Silva PP, Barbosa MH (2022). Effect of listening to music on anxiety, pain, and cardiorespiratory parameters in cardiac surgery: Study protocol for a randomized clinical trial. Trials..

[12] Boswell MR, Moman RN, Burtoft M, Gerdes H, Martinez J, Gerberi DJ, Wittwer E, Murad MH, Hooten WM (2021). Lidocaine for postoperative pain after cardiac surgery: A systematic review. J. Cardiothorac. Surg..

[13] Aktaş YY, Karabulut N (2019). The use of cold therapy, music therapy and lidocaine spray for reducing pain and anxiety following chest tube removal. Complement. Ther. Clin. Pract..

[14] Friesner SA, Curry DM, Moddeman GR (2006). Comparison of two pain-management strategies during chest tube removal: Relaxation exercise with opioids and opioids alone. Heart Lung..

[15] Watanabe SN, Imai K, Kimura T, Saito Y, Takashima S, Matsuzaki I, Kurihara N, Atari M, Matsuo T, Iwai H, Sato Y, Motoyama S, Nomura K, Nishikawa T, Minamiya Y (2019). Effect of lidocaine cream analgesia for chest drain tube removal after video-assisted thoracoscopic surgery for lung cancer: A randomized clinical trial. Reg. Anesth. Pain Med..

[16] Pinheiro VF, da Costa JM, Cascudo MM, Pinheiro Êde O, Fernandes MA, de Araujo IB (2015). Analgesic efficacy of lidocaine and multimodal analgesia for chest tube removal: A randomized trial study. Rev. Lat. Am. Enfermagem..

[17] Ghazali D, Ilha-Schuelter P, Barbosa S, Truchot J, Ceccaldi P, Tourinho F, Plaisance P (2021). Interdisciplinary teamwork for chest tube insertion and management: An integrative review. Anaesthesiol. Intensive Ther..

[18] Hasanzadeh F, Kashouk NM, Amini S, Asili J, Emami SA, Vashani HB, Sahebkar A (2016). The effect of cold application and lavender oil inhalation in cardiac surgery patients undergoing chest tube removal. EXCLI J..

[19] Yarahmadi S, Mohammadi N, Ardalan A, Najafizadeh H, Gholami M (2018). The combined effects of cold therapy and music therapy on pain following chest tube removal among patients with cardiac bypass surgery. Complement. Ther. Clin. Pract..

[20] Jarrah MI, Hweidi IM, Al-Dolat SA, Alhawatmeh HN, Al-Obeisat SM, Hweidi LI, Hweidi AI, Alkouri OA (2022). The effect of slow deep breathing relaxation exercise on pain levels during and post chest tube removal after coronary artery bypass graft surgery. Int. J. Nurs. Sci..

[21] Snoswell AJ, Snoswell CL (2019). Immersive virtual reality in health care: Systematic review of technology and disease states. JMIR Biomed. Eng..

[22] Ebrahimi H, Namdar H, Ghahramanpour M, Ghafourifard M, Musavi S (2017). Effect of virtual reality method and multimedia system on burn patients’ pain during dressing. J. Clin. Anal. Med..

[23] Ford CG, Manegold EM, Randall CL, Aballay AM, Duncan CL (2018). Assessing the feasibility of implementing low-cost virtual reality therapy during routine burn care. Burns..

[24] Chad R, Emaan S, Jillian O (2018). Effect of virtual reality headset for pediatric fear and pain distraction during immunization. Pain Manag..

[25] Won AS, Bailey J, Bailenson J, Tataru C, Yoon IA, Golianu B (2017). Immersive virtual reality for pediatric pain. Children (Basel)..

[26] Gold JI, Mahrer NE (2018). Is virtual reality ready for prime time in the medical space? A randomized control trial of pediatric virtual reality for acute procedural pain management. J. Pediatr. Psychol..

[27] Scapin S, Echevarría-Guanilo ME, Boeira Fuculo Junior PR, Gonçalves N, Rocha PK, Coimbra R (2018). Virtual reality in the treatment of burn patients: A systematic review. Burns..

[28] Indovina P, Barone D, Gallo L, Chirico A, De Pietro G, Giordano A (2018). Virtual reality as a distraction intervention to relieve pain and distress during medical procedures: A comprehensive literature review. Clin. J. Pain..

[29] Sessler CN, Gosnell MS, Grap MJ, Brophy GM, O'Neal PV, Keane KA, Tesoro EP, Elswick RK (2002). The Richmond Agitation-Sedation Scale: Validity and reliability in adult intensive care unit patients. Am. J. Respir. Crit. Care Med..

[30] Kang M, Ragan BG, Park JH (2008). Issues in outcomes research: An overview of randomization techniques for clinical trials. J. Athl. Train..

[31] Gift AG (1989). Visual analogue scales: Measurement of subjective phenomena. Nurs. Res..

[32] Shaban M, Rasoolzadeh N, Mehran A, Moradalizadeh F (2006). Study of two non-pharmacological methods, progressive muscle relaxation and music, on pain relief of cancerous patients. J. Hayat..

[33] Lovibond PF, Lovibond SH (1996). Depression anxiety and stress scales. Behav. Res. Ther..

[34] Norton PJ (2007). Depression Anxiety and Stress Scales (DASS-21): Psychometric analysis across four racial groups. Anxiety Stress Coping.

[35] Asghari A, Saed F, Dibajnia P (2008). Psychometric properties of the Depression Anxiety Stress Scales-21 (DASS-21) in a non-clinical Iranian sample. Int. J. Psychol..

[36] Pickard-Holley S (1991). Fatigue in cancer patients: A descriptive study. Cancer Nurs..

[37] Bahrami T, Rejeh N, Heravi-Karimooi M, Vaismoradi M, Tadrisi SD, Sieloff CL (2018). Aromatherapy massage versus reflexology on female elderly with acute coronary syndrome. Nurs. Crit. Care..

[38] Ioannou A, Papastavrou E, Avraamides MN, Charalambous A (2020). Virtual reality and symptoms management of anxiety, depression, fatigue, and pain: A systematic review. SAGE Open Nurs..

[39] Özkan ZK, Gökçe Işıklı A, Yanık F (2023). The effect of virtual reality glasses on reducing pain during chest tube removal. J. Clin. Med. Kaz..

[40] Orhan M, Bülez A (2023). The effect of virtual reality glasses applied during the episiotomy on pain and satisfaction: A single blind randomized controlled study. J. Pain Res..

[41] Menekli T, Yaprak B, Doğan R (2022). The effect of virtual reality distraction intervention on pain, anxiety, and vital signs of oncology patients undergoing port catheter implantation: A randomized controlled study. Pain Manag. Nurs..

[42] Karaveli Çakır S, Evirgen S (2021). The effect of virtual reality on pain and anxiety during colonoscopy: A randomized controlled trial. Turk. J. Gastroenterol..

[43] Bouraghi H, Mohammadpour A, Khodaveisi T, Ghazisaeedi M, Saeedi S, Familgarosian S (2023). Virtual reality and cardiac diseases: A systematic review of applications and effects. J. Healthc. Eng..

[44] Kodvavi MS, Asghar MA, Ghaffar RA, Nadeem I, Bhimani S, Kumari V, Rabbani A, Iqbal M, Naeem R, Nasir AM, Hassan SS, Ghazni MS (2023). Effectiveness of virtual reality in managing pain and anxiety in adults during periprocedural period: A systematic review and meta-analysis. Langenbecks Arch. Surg..

[45] Chan E, Foster S, Sambell R, Leong P (2018). Clinical efficacy of virtual reality for acute procedural pain management: A systematic review and meta-analysis. PLoS One..

[46] Karaman D, Taşdemir N (2021). The effect of using virtual reality during breast biopsy on pain and anxiety: A randomized controlled trial. J. Perianesth. Nurs..

[47] Vasquez JM, Vaca VL, Wiederhold BK, Miller I, Wiederhold MD (2017). Virtual reality pain distraction during gynecological Surgery—A report of 44 cases. Surg. Res. Updates..

[48] Guo C, Deng H, Yang J (2015). Effect of virtual reality distraction on pain among patients with hand injury undergoing dressing change. J. Clin. Nurs..

[49] Glennon C, McElroy SF, Connelly LM, Mische Lawson L, Bretches AM, Gard AR, Newcomer LR (2018). Use of virtual reality to distract from pain and anxiety. Oncol. Nurs. Forum..

[50] Ridout B, Kelson J, Campbell A, Steinbeck K (2021). Effectiveness of virtual reality interventions for adolescent patients in hospital settings: Systematic review. J. Med. Int. Res..

[51] Karami M, Gohari AR, Ebrahimzadeh MA, Danaei GH (2016). Pathophysiology of pain and effective drugs to control it. Clin. Excell..

[52] Hyndman, A., Sauriol, N., inventors & Avaya Inc., assignee. Method and apparatus for monitoring user attention with a computer-generated virtual environment. United States patent US 8,542,232. 2013.

[53] Christensen, J., Annau, T. M., Van Hoff, A., inventors & Verizon Patent, Licensing Inc, assignee. Generating content for a virtual reality system. United States patent US 10,708,568. 2020.

[54] Crombez G, Veirman E, Van Ryckeghem D, Scott W, De Paepe A (2023). The effect of psychological factors on pain outcomes: Lessons learned for the next generation of research. Pain Rep..

